# Supplementary material for: Mirtron-mediated RNA knockdown/replacement therapy for the treatment of dominant retinitis pigmentosa
Source: Nat Commun. 2021 Aug 16;12:4934. doi: 10.1038/s41467-021-25204-3 (PMC8368061; doi:10.1038/s41467-021-25204-3)
Supplement: Supplementary file 1 — Supplementary Information [file 41467_2021_25204_MOESM1_ESM.pdf]

Mirtron-mediated RNA knockdown/replacement therapy for the treatment of dominant retinitis pigmentosa: Supplementary material

*Harry O. Orlans[1,2] , Michelle E. McClements[1], Alun R. Barnard[1], Cristina Martinez-Fernandez de la Camara[1], Robert E. MacLaren[1,2]

[1]Nuffield Laboratory of Ophthalmology, Level 6, West Wing, John Radcliffe Hospital, Headley Way, Headington, Oxford OX3 9DU, UK

[2]Moorfields Eye Hospital, 162 City Road, London EC1V 2PD, UK

*Corresponding author:

Harry Orlans

Nuffield Laboratory of Ophthalmology, Level 6, West Wing, John Radcliffe Hospital, Headley Way, Headington, Oxford OX3 9DU

Email: Enquiries@eye.ox.ac.uk

Tel: 0044 (0)1865 234 829

Supplementary note 1. Assessment of potential off-target effects

If mirtrons are to be used for the treatment of patients with RP, it is important that off-target effects within transduced rods are minimal. To investigate this, the 21bp target sequences for the three most effective mirtrons (M2, M3 and M5) were subjected to a BLAST search (www.endembl.org) for homology within the mouse and human transcriptomes. All matches with an E-value (the calculated probability of the alignment between the input and subject sequences being attributable to chance) of less than ten were considered (Supplementary Fig. 1a). Potential targets for the passenger strand sequences of these mirtrons were also considered and are shown in Supplementary Fig. 1b. The longest guide strand off-target match was an 18bp complementary sequence (with one internal mismatch) within the GPT2 coding sequence (CDS) (E-value=5.5). Several 14bp alignments within the human transcriptome were also identified for M3 and 5H which included a match between the M3 guide sequence and the 3'-UTR of the SEC14L2 gene (E-value=5.5). This potential target (along with GPT2) was explored further, as expression within rod outer segment has been documented[1]. To quantify the extent to which these predicted sequences are true off-targets for M3, the target sequence for each (along with 25bp of upstream and downstream flanking DNA) was ligated into the multiple cloning site of the PsiCHECK2 vector (Promega, UK). The subsequent Dual Glo® assay performed against M3 suggested that mRNA for GPT2 and SEC14L2 are not subjected to M3-mediated knockdown (Supplementary Fig. 1c). It should be noted that miRNA off-targets in the 3'-UTRs are particularly associated with the seed region comprising positions 2-7 of the mature miRNA[2]. Full analysis of the transcripts meeting this criterion is beyond the scope of the current study but would be an important area of investigation prior to clinical translation of a mirtron-based therapy.

a

Mirtron	Human targets	Match (bp)	E-value	Mouse targets	Match (bp)	E-value
2	*RHO*	21	0.0004	*Rho*	21	0.0002
				Tenm2	14	3.7
3	*RHO*	21	0.0004	*Rho*	21	0.0002
	GPT2	18†	5.5	*Ctsc*	14	3.7
	IL34	14	5.5	*Lgals4*	14	3.7
	PTF1A	14	5.5	*Tenm2*	14	3.7
	*SEC14L2**	14	5.5			
	GOLGA8B	14	5.5			
	AKNA	14	5.5			
	INPL1	14	5.5			
5^H	*RHO*	21	0.0004	*Rho*	21	0.0002
	ZBTB10	14	5.5	*Ptprb*	15	0.94
	RHPT1	14	5.5	*Tgm7*	14	3.7
	RHOT1	14	5.5	*Mtm*	14	3.7
	C11orf40	14	5.5	*Mfsd8*	14	3.7
				Ikbkap	14	3.7
5^M	None	-	-	*Rho*	21	0.0003
				Pnn	14	3.9
				Dync1b1	14	3.9
				Erg	14	3.9

b

Mirtron	Human targets	Match (bp)	E-value	Mouse targets	Match (bp)	E-value
2	*SYNDIG1L*	15	1.4	*Swap70*	15	0.98
	TENM2	14	5.7			
3	*LAMA2*	15	1.4	*Olfr1384*	16	0.24
	GOLGA8N	14	5.5	*Gpr139*	16	0.24
	CES2	14	5.5	*Olfr281*	14	3.7
	CWC22	14	5.5	*Il34*	14	3.7
	SERPIND1	14	5.5			
	PTPN9	14	5.5			
5^H	*ADAMTSL1*	18	0.023	*Olfr715*	15	0.94
	DRAXIN	15	1.4	*Mcpb1*	15	0.94
	ZNF326	14	5.5	*Ndufaf4*	14	3.7
	HERC1	14	5.5			
	JPH1	14	5.5			
	RXFP1	14	5.5			
	FGF18	14	5.5			
5^M	*ASPA*	16	0.36	None	-	-
	CHST9	14	5.7			
	FSHB	14	5.7			
	ZNF624	14	5.7			
	ZNF480	14	5.7			
	GCLC	14	5.7			
	GMNC	14	5.7			

c

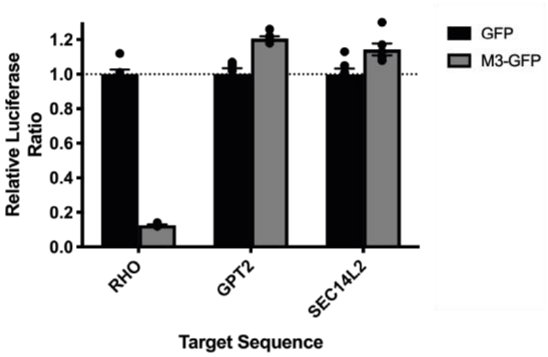

Supplementary Fig. 1. Potential off-targets of rhodopsin-targeting artificial mirtrons. a, Predicted off-targets for the guide strand of mirtrons 2, 3 and 5. **b,** Predicted off-targets for the passenger strand of mirtrons 2, 3 and 5. In both cases the 21bp guide/passenger sequences were input into BLAST searches of the human and mouse cDNA databases (transcripts/splice variants). *Gene expression previously described in photoreceptors. †One internal mismatch. **c,** Mirtron 3 does not target the predicted off-target mRNA transcripts of the human *GPT2* and *SEC14L2* genes. Target sequences within *RHO*, *GPT2* and *SEC14L2* were cloned into the PsiCHECK2 vector and a Dual Glo® assay performed. Bars represent mean ± SEM, n=6.

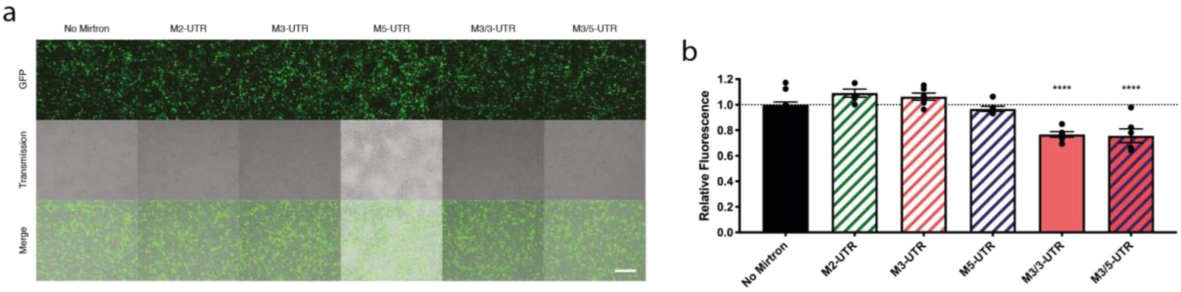

Supplementary Fig. 2. Effect of mirtrons located within the 5'-UTR on downstream transgene expression. a, Fluorescence micrographs of HEK293 cells following transfection with 5'-UTR-GFP expression plasmids. Scale bar: 200μm; n=6. **b,** Fluorescence spectroscopy assay using transfected HEK293 cell lysates (mean ± SEM; n=6). Values are normalised to fluorescence levels recorded form cells transfected with the mirtronless GFP plasmid. ****$p<0.0001$, ordinary one-way ANOVA.

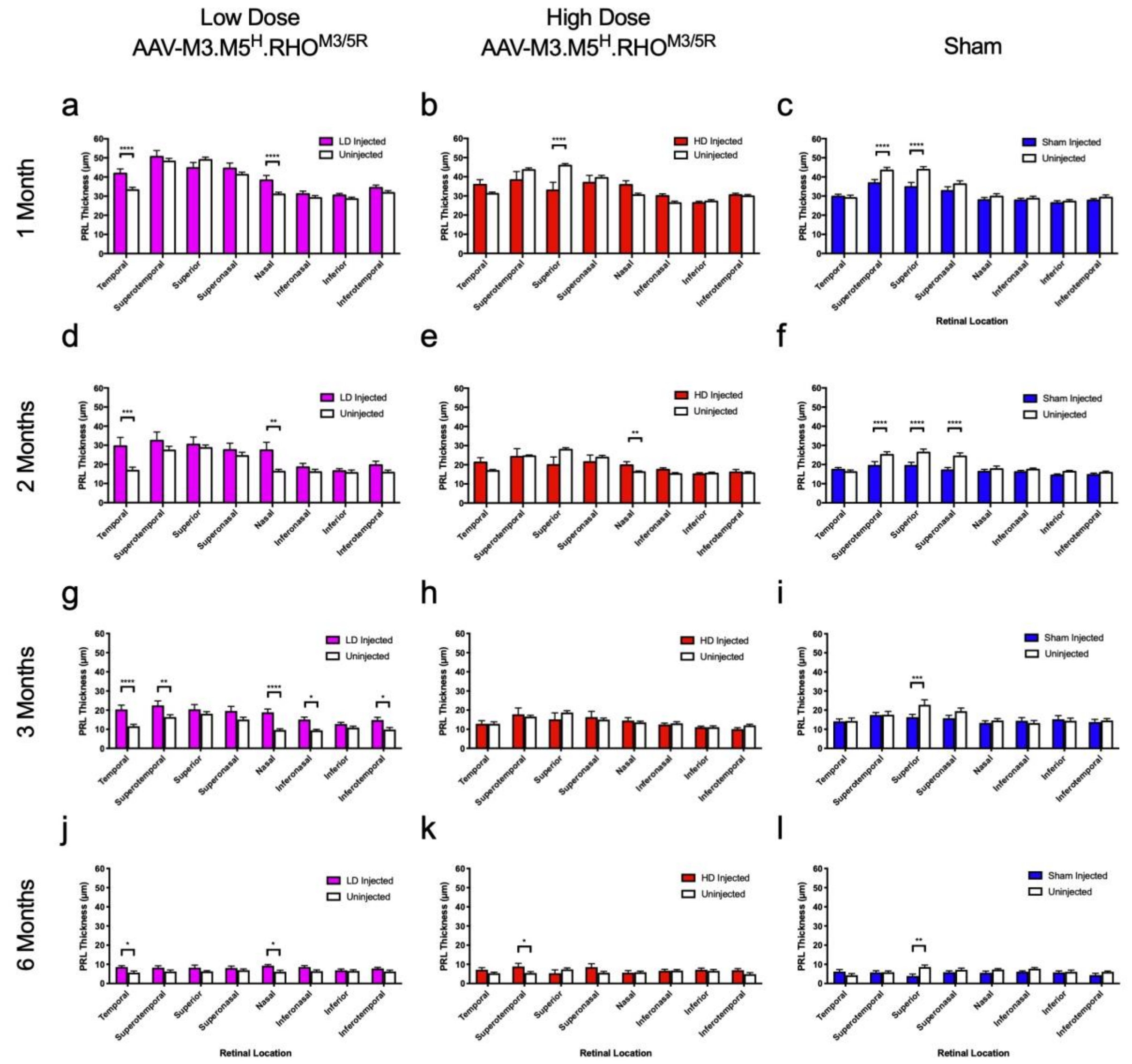

Supplementary Fig. 3. Effect of subretinal injection of AAV-M3.M5^{H}.RHO$^{M3/5R}$ on mean PRL thickness by retinal location in the *Nrl.GFP/+, Rho$^{P23H/+}$* mouse. All measurements were taken at a fixed eccentricity of approximately 22.5° from the optic nerve head in the stated direction. **a, d, g, j,** Low dose (2x10^{8} gc) AAV-M3.M5^{H}.RHO$^{M3/5R}$-injected cohort at 1 (a), 2 (d), 3 (g) and 6 (j) months post-injection (n=17). **b, e, h, k,** High dose (2x10^{9} gc) AAV-M3.M5^{H}.RHO$^{M3/5R}$-injected cohort at 1 (b), 2 (e), 3 (h) and 6 (k) months post-injection (n=17). **c, f, i, l,** Sham (PBS)-injected cohort at 1 (c), 2 (f), 3 (i) and 6 (l) months post-injection (n=14). Data plotted as mean ± SEM throughout. *p<0.05; **p<0.01; ***p<0.001; ****p<0.0001, all other comparisons not statistically significant, two-sided Šidák's multiple comparison test.

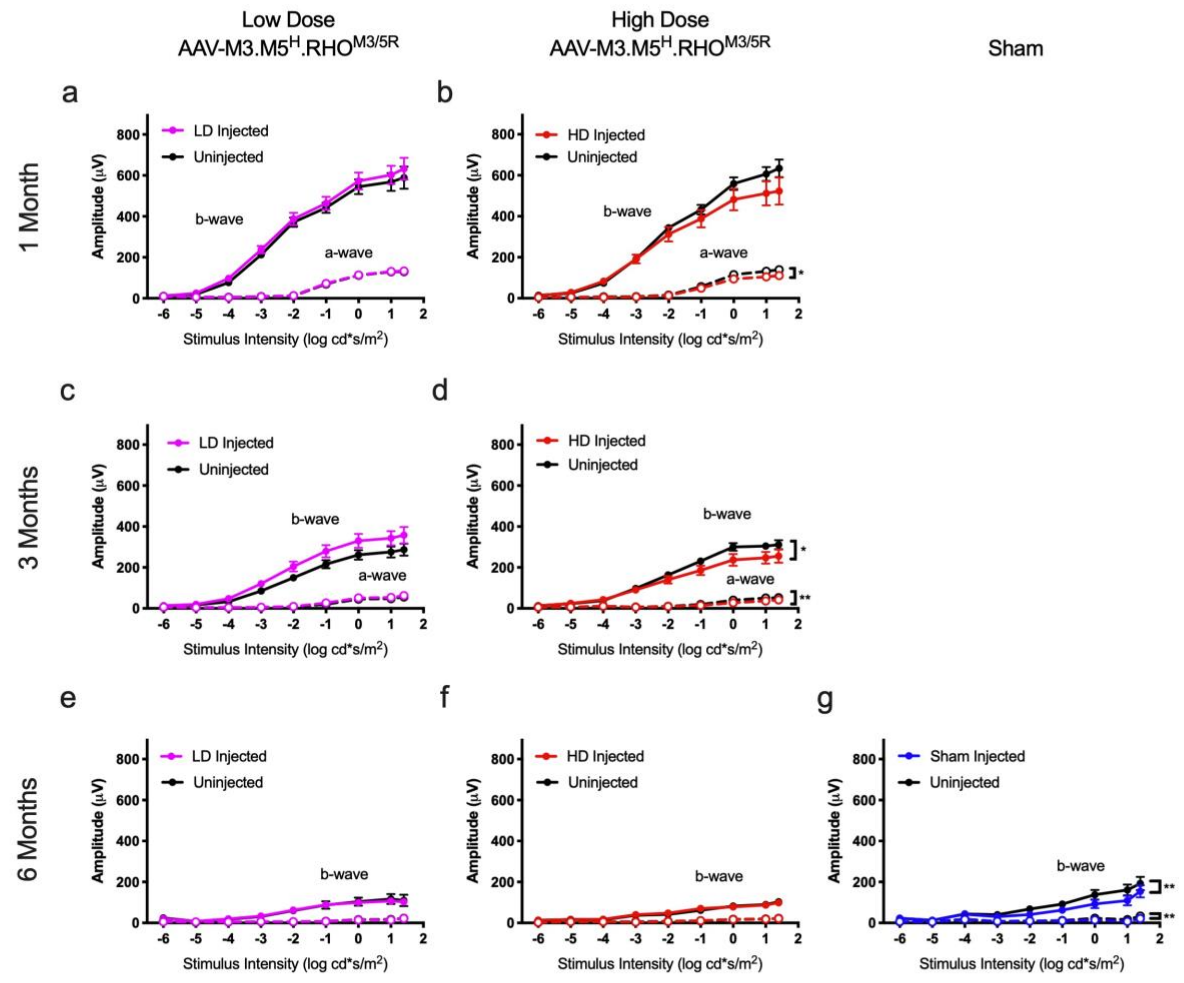

Supplementary Fig. 4. Full field dark-adapted ERG irradiance-response curves for low and high dose AAV-M3.M5^{H}.RHO$^{M3/5R}$-injected and sham-injected cohorts. All data points represent mean signal ± SEM. Separate curves are plotted for injected right eyes and uninjected left eyes for all cohorts. **a, c, e,** Low dose (LD) AAV-M3.M5^{H}.RHO$^{M3/5R}$-injected cohort, n=17. **b, d, f,** High dose (HD) AAV-M3.M5^{H}.RHO$^{M3/5R}$-injected cohort, n=17. One month a-wave *p=0.042; 3 months a-wave **p=0.004; 3 months b-wave *p=0.042. **g,** Sham (PBS)-injected cohort, n=14; a-wave **p=0.0022; b-wave **p=0.0021. Data for sham cohort at months one and three has been presented elsewhere[3]. Repeated measures two-way ANOVA for effect of injection.

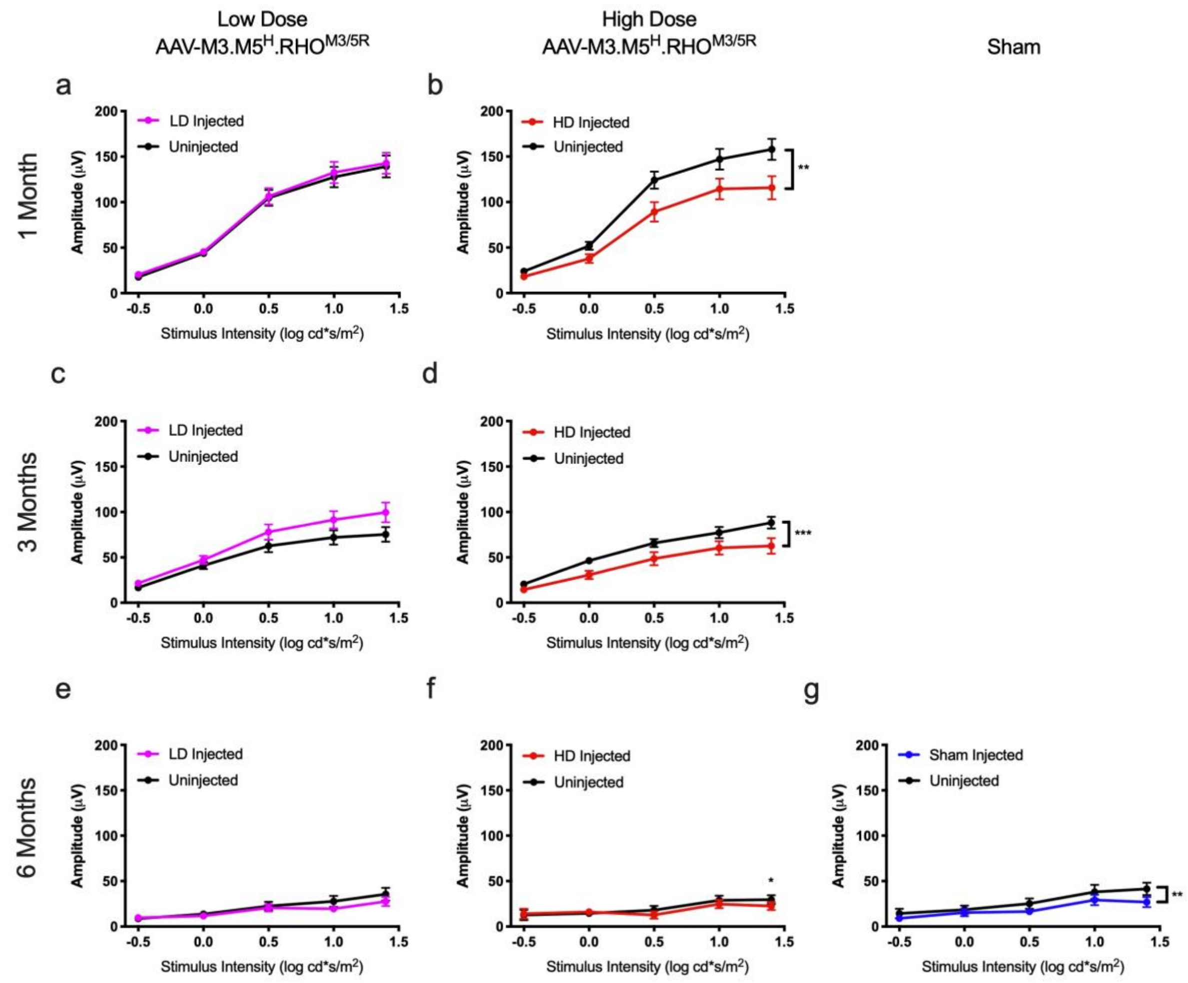

Supplementary Fig. 5. Full field light-adapted ERG irradiance-response curves for low and high dose AAV-M3.M5^{H}.RHO$^{M3/5R}$-injected and sham-injected cohorts. All data points represent mean signal ± SEM. Separate curves are plotted for injected right eyes and uninjected left eyes for all cohorts. **a, c, e,** Low dose (LD) M3.M5^{H}.RHO$^{M3/5R}$-injected cohort, n=17. **b, d, f,** High dose (HD) M3.M5^{H}.RHO$^{M3/5R}$-injected cohort, n=17; *p=0.019; **p=0.0058; ***p=0.0007. **g,** Sham (PBS)-injected cohort, n=14; p=0.0058. Data for sham cohort at months one and three has been presented elsewhere[3]. Repeated measures two-way ANOVA for effect of injection.

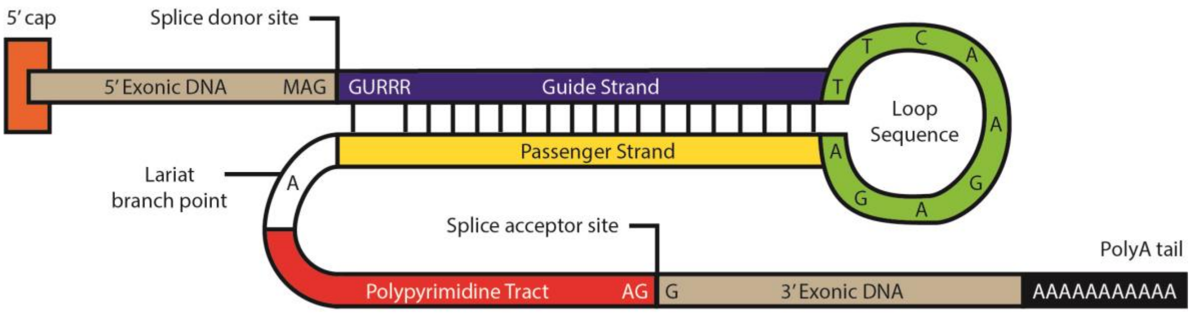

Supplementary Fig. 6. General structure of 3' tailed mirtrons. The schematic illustrates a 3' tailed mirtron as an intron within a pre-mRNA transcript. The mirtron itself consists of (from 5' to 3'): guide strand, loop sequence (TTCAAGAGA), passenger strand, lariat branch point (A), and polypyrimidine tract. The mirtron commences with a 5' splice donor motif and ends with the 3' splice acceptor motif. All mirtrons used in this study were 76bp in length. The guide and passenger strands (21bp in length) form a hairpin structure through Watson-Crick base pair complementarity, with mismatches being purposefully introduced at the base of the stem to facilitate guide strand selection by the RISC. M = A or C; R = A or G.

	Spliceport		Human Splice Finder	
Mirtron Design	Donor	Acceptor	Donor	Acceptor
1	0.26	1.60	75.21	86.7
2	0.9	2.38	76.61	86.7
3	1.83	2.13	95.95	86.7
4	0.41	2.06	83.94	86.7
5H	0.95	1.84	78.54	86.7
5M	1.12	1.93	78.54	86.7
6	0.79	2.16	83.79	86.7
7	2.15	2.19	95.71	86.7

Supplementary Table 1. Splice donor and acceptor site strength prediction for artificial mirtron designs. The CDS for *eGFP* with artificial mirtrons nested at the BstbI cut site were input into Spliceport and Human Splice Finder online software. Higher scores in both programmes denote a greater likelihood of the intended splice donor and acceptor splice sites being recognised as such by cellular splice machinery.

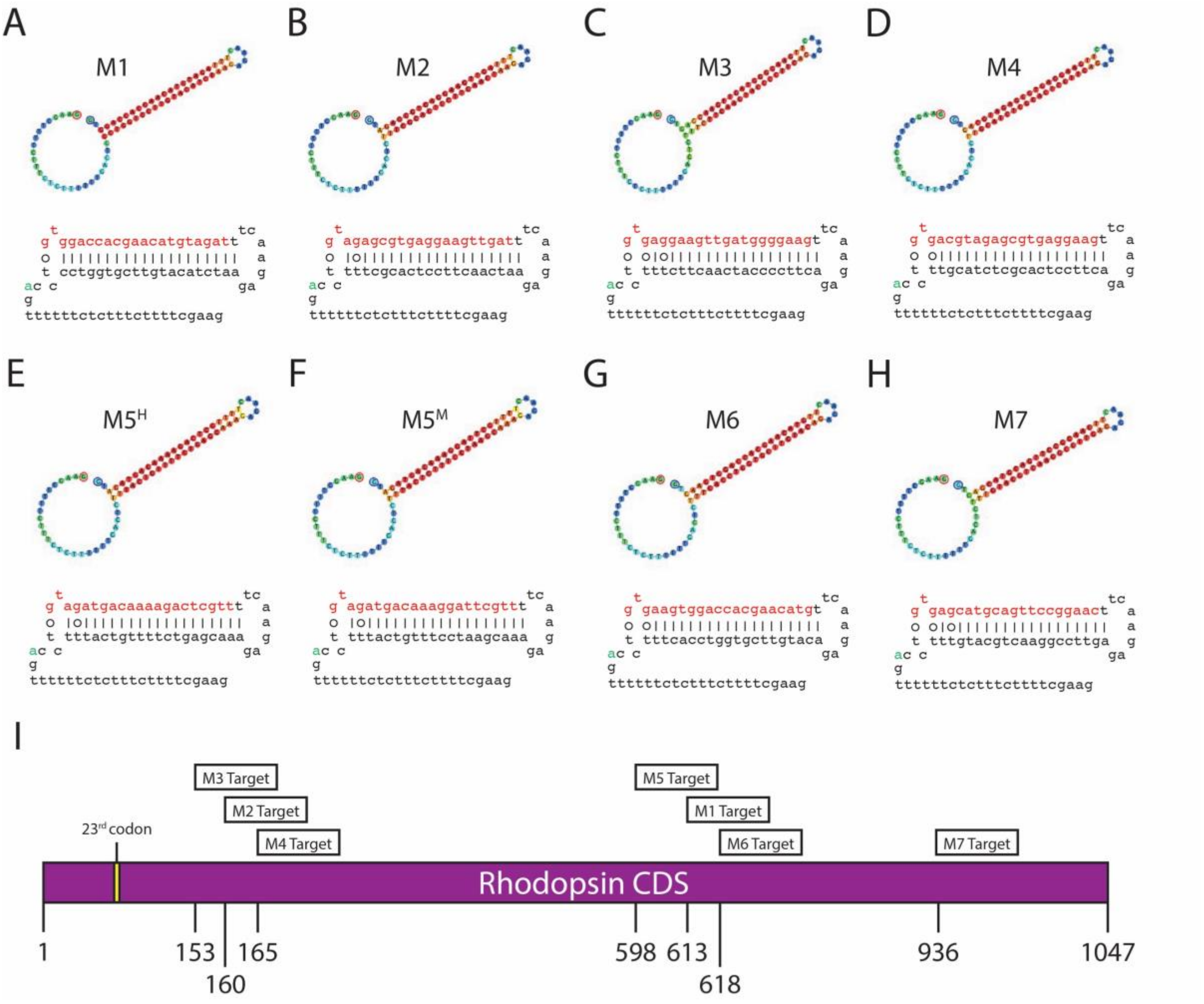

Supplementary Fig. 7. Mirtron designs. (A-H) RNA fold diagrams (upper panels) and sequences (lower panels) of the 76bp structures of Mirtrons 1-7. RNA secondary structures are based on the probabilistic energy model described by Matthews et al., 2004[4]. Thermodynamic stability of predicted base pairing is indicated by colour with red representing the highest probability and blue the lowest. The 5' and 3' ends are indicated by blue and red circles respectively. Note that in all cases, the predicted internal stability at the base of the stem structure is lower than that of the distal end near the loop. This design feature was intended to favour selection of the guide (5') strand by the RISC[5]. Images generated using Geneious software (v11.0.2, Biomatters Ltd., Auckland, New Zealand). In the sequence diagrams, the guide strand is shown in red whilst the predicted lariat branch point is highlighted in green. **(I)** Relative positions of the mirtron target sequences within the rhodopsin CDS (source: NCBI consensus CDS database reference CCDS3063.1). The 1047bp rhodopsin coding sequence is represented by a purple box. The relative locations of the 21bp target sequences for mirtrons 1 to 7 are indicated above the RHO CDS whilst the position of the first nucleotide of each target is shown beneath it. The position of the 23rd codon is indicated by a yellow box and was not targeted by any of the seven mirtron designs. Schematic not to scale.

Mirtron	Guide Strand Sequence	Target Sequence	Target Position
1	GTGGACCACGAACATGTAGAT	ATCTACATGTTCGTGGTCCAC	613-633
2	GTAGAGCGTGAGGAAGTTGAT	ATCAACTTCCTCACGCTCTAC	160-180
3	GTGAGGAAGTTGATGGGGAAG	CTTCCCCATCAACTTCCTCAC	153-173
4	GTGACGTAGAGCGTGAGGAAG	CTTCCTCACGCTCTACGTCAC	165-185
5H	GTAGATGACAAAAGACTCGTT	AACGAGTCTTTTGTCATCTAC	598-618
5M	GTAGATGACAAAGGATTCGTT	AACGAATCCTTTGTCATCTAC	598-618
6	GTGAAGTGGACCACGAACATG	CATGTTCGTGGTCCACTTCAC	618-638
7	GTGAGCATGCAGTTCCGGAAC	GTTCCGGAACTGCATGCTCAC	936-956

Supplementary Table 2. Mirtron guide and target sequences. The 21bp guide strands and corresponding target sequences within rhodopsin for mirtrons 1-7 are displayed. The position of each target sequence within the rhodopsin CDS in bp is shown in the right-hand column.

Primer Name	Primer Use	Primer Sequence
**Mirt FW**	Mirtron cloning	5'- CACAAATTTTCTGTCAGC
**XhoI RHO FW**	Mirtron cloning	5'- ATCTCGAGATGAATGGCACAGA
**RHO NotI RC**	Mirtron cloning	5'- ATGCGGCCGCTTAGGCCGGGGCCA
**AsiSI mRho FW**	Mirtron cloning	5'-ATGCGATCGCATGAACGGCACAGA
**mRho NotI RC**	Mirtron cloning	5'-ATGCGGCCGCTTAGGCTGGAGCCA
**PC Seq FW**	Mirtron cloning	5'- TCGAGTCCGACCCTGGGTTCT

**Mirt Splice FW**	Mirtron splice assay	5’-GACGGGAACTACAAGACCCG
**Mirt Splice RC**	Mirtron splice assay	5’-CTGCACGGATCCATCCTCAA
**UTR-Mirt FW**	5’-UTR mirtron plasmid cloning	5’- TGACGGGAACTACAAGACCC
**UTR-Mirt RC**	5’-UTR mirtron plasmid cloning	5’- ATCCTCCTTAAAGTCAATGCCC
**UTR Splice FW**	5’-UTR mirtron splice assay	5’-GCTGGTTATTGTGCTGTCTC
**UTR Splice RC**	5’-UTR mirtron splice assay	5’-GGGCTGCAGGAATTCGAT
**M3CO-RHO FW**	Codon-modified RHO cloning	5’- ATTTCCAATTAATTTTCTGACGCTCTACGTCACCG
**M3CO-RHO RC**	Codon-modified RHO cloning	5’ CAGAAAATTAATTGGAAATCCCAGCACGATCAGCAG
**M5CO-RHO FW**	Codon-modified RHO cloning	5’- TGAATCCTTCGTGATTTATATGTTCGTGGTCCAC
**M5CO-RHO RC**	Codon-modified RHO cloning	5’- ATAAATCACGAAGGATTCATTGTTGACCTCCGGC

**EcoRI RHO FW**	Mirtron AAV plasmid cloning	5'-TCGAATTCCGCCACCATGAATGGCAC
**RHO Splice RC**	In vivo splice assay	5'- CCAGCACGATCAGCAGAAAC

Supplementary Table 3. Sequences for primers used in this study.

Upstream flank size (bp)	Donor site score	Downstream flank size (bp)	Acceptor site score
**0**	-1.01	0	2.41
**5**	0.8	5	3.02
**10**	0.59	10	2.65
**15**	0.4	15	2.51
**20**	1.07	20	2.32
**25**	1.21	25	2.26
**30**	0.92	30	2.1
**35**	1.56	35	2.47
**41**	1.73	40	2.27
		45	2.06
		50	2.35
		53	2.55

Supplementary Table 4. In silico optimisation of mirtron flank sequence length. Having determined that mirtrons splice effectively from the BstbI restriction site within the *eGFP* CDS, an in silico analysis was performed to determine the optimal likely upstream and downstream flanking sequence length that would ensure this splicing efficiency is maintained when cloned into the 5'-UTR. Scores >0 give a false positive rate for splice donor and acceptor sites of 1.41% and 1.54% respectively whilst scores >1 give a false positive rate of 0.09% and 0.12% (Spliceport online tool; http://spliceport.cbcb.umd.edu).

Upstream flank:

```
           ACAAGA                 TTCGAA
          TACAAG            GTCAAG
        ACTACA          TGAAGT
       AACTAC          CTGAAG
TGACGGGAACTACAAGACCCGCGCTGAAGTCAAGTTCGAAG
```

Downstream flank:

```
                                               GAGGAT
                           TGAAGG            GGAGGA
                          CTGAAG           AAGGAG
GTGACACCCTGGTGAATAGAATCGAGCTGAAGGGCATTGACTTTAAGGAGGAT
```

Supplementary Fig. 8. Upstream (5') and downstream (3') mirtron flank sequences. Flank sequences are shown in black above which the positions of ESE hexamer motifs are indicated in red.

Primary antibody target	Clonality/Epitope	Reference	Secondary antibody	Reference
Rhodopsin	Polyclonal	ab3424	Alexa-fluor donkey anti-rabbit IgG 568	ab175470
Rhodopsin	Monoclonal/ 1D4 (C-terminus)	ab5417	Alexa-fluor donkey anti-mouse IgG 568	ab175472
Rhodopsin	Monoclonal/ 4D2 (N-terminus)	ab98887	Alexa-fluor donkey anti-mouse IgG 568	ab175472

Supplementary Table 5. Antibodies used for immunocytochemistry. All antibodies were manufactured by Abcam, UK. Primary antibodies were diluted 1:1000, whilst secondary antibodies were diluted 1:200.

Supplementary Material References

1. Datta, P. *et al.* Accumulation of non-outer segment proteins in the outer segment underlies photoreceptor degeneration in Bardet-Biedl syndrome. *Proc. Natl. Acad. Sci. U. S. A.* **112,** E4400–E4409 (2015).

2. Haley, B. & Zamore, P. D. Kinetic analysis of the RNAi enzyme complex. *Nat. Struct. Mol. Biol.* **11**, 599–606 (2004).

3. Orlans, H., Barnard, A. R., Patricio, M. M., McClements, M. E. & MacLaren, R. E. Effect of AAV-mediated rhodopsin gene augmentation on retinal degeneration caused by the dominant P23H rhodopsin mutation in a knock-in murine model. *Hum. Gene Ther.* (2020). doi:10.1089/hum.2020.008

4. Mathews, D. H. *et al.* Incorporating chemical modification constraints into a dynamic programming algorithm for prediction of RNA secondary structure. *Proc. Natl. Acad. Sci. U. S. A.* **101**, 7287 LP – 7292 (2004).

5. Reynolds, A. *et al.* Rational siRNA design for RNA interference. *Nat. Biotechnol.* **22**, 326–330 (2004).
