# Supplementary material for: Mirtron-mediated RNA knockdown/replacement therapy for the treatment of dominant retinitis pigmentosa
Source: Nat Commun. 2021 Aug 16;12:4934. doi: 10.1038/s41467-021-25204-3 (PMC8368061; doi:10.1038/s41467-021-25204-3)
Supplement: Supplementary file 3 — Reporting Summary [file 41467_2021_25204_MOESM3_ESM.pdf]

nature research

Corresponding author(s): Harry Orlans

Last updated by author(s): Jul 13, 2021

Reporting Summary

Nature Research wishes to improve the reproducibility of the work that we publish. This form provides structure for consistency and transparency in reporting. For further information on Nature Research policies, see our Editorial Policies and the Editorial Policy Checklist.

Statistics

For all statistical analyses, confirm that the following items are present in the figure legend, table legend, main text, or Methods section.

n/a	Confirmed	
☐	☒	The exact sample size (*n*) for each experimental group/condition, given as a discrete number and unit of measurement
☐	☒	A statement on whether measurements were taken from distinct samples or whether the same sample was measured repeatedly
☐	☒	The statistical test(s) used AND whether they are one- or two-sided<br>*Only common tests should be described solely by name; describe more complex techniques in the Methods section.*
☐	☒	A description of all covariates tested
☐	☒	A description of any assumptions or corrections, such as tests of normality and adjustment for multiple comparisons
☐	☒	A full description of the statistical parameters including central tendency (e.g. means) or other basic estimates (e.g. regression coefficient) AND variation (e.g. standard deviation) or associated estimates of uncertainty (e.g. confidence intervals)
☐	☒	For null hypothesis testing, the test statistic (e.g. *F*, *t*, *r*) with confidence intervals, effect sizes, degrees of freedom and *P* value noted<br>*Give P values as exact values whenever suitable.*
☒	☐	For Bayesian analysis, information on the choice of priors and Markov chain Monte Carlo settings
☒	☐	For hierarchical and complex designs, identification of the appropriate level for tests and full reporting of outcomes
☒	☐	Estimates of effect sizes (e.g. Cohen's *d*, Pearson's *r*), indicating how they were calculated

Our web collection on statistics for biologists contains articles on many of the points above.

Software and code

Policy information about availability of computer code

Data collection	All software has been referenced appropriately within the manuscript or references included to previous studies in which this information has been provided.
Data analysis	Graphpad Prism software version 7.0a. Human Splice Finder Version 3.1. Spliceport: http://spliceport.cbcb.umd.edu.

For manuscripts utilizing custom algorithms or software that are central to the research but not yet described in published literature, software must be made available to editors and reviewers. We strongly encourage code deposition in a community repository (e.g. GitHub). See the Nature Research guidelines for submitting code & software for further information.

Data

Policy information about availability of data

All manuscripts must include a data availability statement. This statement should provide the following information, where applicable:

- Accession codes, unique identifiers, or web links for publicly available datasets
- A list of figures that have associated raw data
- A description of any restrictions on data availability

Raw data that support the findings in this study will be deposited in the Oxford Research Archive (ORA: https://ora.ox.ac.uk). Additionally data can be provided by the corresponding author upon request.

Field-specific reporting

Please select the one below that is the best fit for your research. If you are not sure, read the appropriate sections before making your selection.

☒ Life sciences ☐ Behavioural & social sciences ☐ Ecological, evolutionary & environmental sciences

For a reference copy of the document with all sections, see nature.com/documents/nr-reporting-summary-flat.pdf

Life sciences study design

All studies must disclose on these points even when the disclosure is negative.

Sample size	Power calculations were performed for all animal experiments to determine sample size. Sample sizes for in vitro experiments were based on numbers used in similar studies e.g. Kock et al 2015 and Cutis et al 2017.
Data exclusions	No data was excluded from this study
Replication	All positive findings in this study were replicable. The number of replicates for any given experiment has been stated within the manuscript where appropriate.
Randomization	Mice were randomly allocated to experimental groups throughout the study.
Blinding	In vitro experiments were always performed with relevant positive and negative controls and across multiple biological replicates in order to achieve reliable data. It was considered that the in vitro data are less prone to bias due to the nature of the measurements taken. All raw data can be made available should there be any concerns. Measures of retinal thickness by OCT and ERG analysis were performed in a blinded fashion in all cases.

Reporting for specific materials, systems and methods

We require information from authors about some types of materials, experimental systems and methods used in many studies. Here, indicate whether each material, system or method listed is relevant to your study. If you are not sure if a list item applies to your research, read the appropriate section before selecting a response.

Materials & experimental systems

n/a	Involved in the study	
☐	☒	Antibodies
☐	☒	Eukaryotic cell lines
☒	☐	Palaeontology and archaeology
☐	☒	Animals and other organisms
☒	☐	Human research participants
☒	☐	Clinical data
☒	☐	Dual use research of concern

Methods

n/a	Involved in the study	
☒	☐	ChIP-seq
☒	☐	Flow cytometry
☒	☐	MRI-based neuroimaging

Antibodies

Antibodies used

1. Anti-rhodopsin antibody ab3424
Rabbit polyclonal to rhodopsin
Manufacturer: Abcam
Catalogue number: ab3424

2. Anti-rhodopsin antibody [1D4]
Mouse monoclanal to rhodopsin
Manufacturer: Abcam
Catalogue number: ab5417

3. Anti-rhodopsin antibody [4D2]
Mouse monoclanal to rhodopsin
Manufacturer: Abcam
Catalogue number: ab598887

4. Alexa-fluor donkey anti-rabbit IgG 568
Donkey Anti-Rabbit IgG H&L (Alexa Fluor® 568)
Conjugation: Alexa Fluor® 568. Ex: 578nm, Em: 603nm
Manufacturer: Abcam
Catalogue number: ab175470

5. Alexa-fluor donkey anti-mouse IgG 568
Donkey Anti-Mouse IgG H&L (Alexa Fluor® 568)
Conjugation: Alexa Fluor® 568. Ex: 578nm, Em: 603nm
Manufacturer: Abcam
Catalogue number: ab175472

Unfortunately clone names and lot numbers were not recorded at time of use and so this information is unavailable.

Validation

Validation statements from Abcam website:

1. Anti-rhodopsin antibody ab3424
Product name: Anti-Rhodopsin antibody
Description: Rabbit polyclonal to Rhodopsin
Host species: Rabbit
Specificity Detects Rhodopsin from bovine retina samples. Data from Yin J et al., 2012 (PMID 22743318) indicates that in Zebrafish ab3424 appears to recognize Red Opsin rather than Rhodopsin.
Species reactivity: Reacts with: Mouse, Cow
Predicted to work with: Rat, Rabbit, Cat, Dog, Human, Cynomolgus monkey, Chinese hamster, African bush elephant Does not react with: Sheep
Immunogen Synthetic peptide corresponding to Cow Rhodopsin aa 338-348.
Sequence:
SKTETSQVAPA

2. Anti-rhodopsin antibody [1D4]
Product name: Anti-Rhodopsin antibody [1D4]
Description: Mouse monoclonal [1D4] to Rhodopsin
Host species: Mouse
Specificity: ab5417 detects Rhodopsin from human and bovine retinal samples. Data from Yin J et al., 2012 (PMID 22743318) indicates that in Zebrafish ab5417 appears to recognize Red Opsin rather than Rhodopsin.
Tested applications: Suitable for: WB, IHC-P
Species reactivity Reacts with: Mouse, Human
Predicted to work with: Zebrafish
Immunogen Other Immunogen Type corresponding to Bovine Rhodopsin.
Epitope The epitope for this antibody has been localized to the C-terminal nine amino acids of bovine rhodopsin known as the 1D4 epitope.

3. Anti-rhodopsin antibody [4D2]
Product name: Anti-Rhodopsin antibody [Rho 4D2]
Description: Mouse monoclonal [Rho 4D2] to Rhodopsin
Host species: Mouse
Tested applications: Suitable for: Flow Cyt, IHC-Fr, WB
Species reactivity: Reacts with: Mouse, Cow, Human
Immunogen: Recombinant fragment corresponding to Bovine Rhodopsin (N terminal).
Database link: P02699
Positive control Flow Cyt: Y79 cells. IHC-Fr: Mouse retina tissue. WB: A549 cell lysate. Bovine photoreceptor membrane tissue lysate.

4. Alexa-fluor donkey anti-rabbit IgG 568
Product name: Donkey Anti-Rabbit IgG H&L (Alexa Fluor® 568)
Host species: Donkey
Target species: Rabbit
Specificity: This antibody is specific to Rabbit IgG
Tested applications: Suitable for: IHC-Fr, ICC/IF, IHC-P, Flow Cyt, ELISA

5. Alexa-fluor donkey anti-mouse IgG 568
Product name: Donkey Anti-Mouse IgG H&L (Alexa Fluor® 568)
Host species: Donkey
Target species: Mouse
Specificity: This antibody is specific to Mouse IgG
Tested applications Suitable for: IHC-Fr, ELISA, Flow Cyt, ICC/IF, IHC-P

Eukaryotic cell lines

Policy information about cell lines

Cell line source(s)

HEK293 #85120602, Culture Collections, Public Health England

Authentication

HEK293 cells were sourced from a reliable provider (Public Health England: https://www.phe-culturecollections.org.uk/products/celllines/generalcell/detail.jsp?refId=85120602&collection=ecacc_gc), not subsequently authenticated. Appearance and behavior of cells throughout the study were consistent with those expected for this commonly used cell line.

Mycoplasma contamination

Cell lines were tested regularly for mycoplasma: negative in all cases.

Commonly misidentified lines (See ICLAC register)	No commonly misidentified cell lines were used in the study.

Animals and other organisms

Policy information about studies involving animals; ARRIVE guidelines recommended for reporting animal research

Laboratory animals	Mice were housed according to UK Home Office guidelines at 20-24oC ambient temperature and 45-65% humidity. Mice were housed in a 12-hour light-dark cycles with food and water available ad libitum. All animal procedures were approved by local and national authorities and in accordance with the Association for Research in Vision and Ophthalmology guidelines for the humane use of laboratory animals in ophthalmic research.<br><br>Mouse species:<br>The Nrl.GFP/+, Rho-/- mouse line was generated by an intercross between the rhodopsin knockout mouse as described by Humphries et al., 1997 (PMID: 9020854;) and a transgenic mouse line expressing enhanced green fluorescent protein (eGFP) under the control of the neural retina-specific leucine zipper (Nrl) promoter [Tg(Nrl-EGFP)], obtained as a kind gift from Professor A. Swaroop, Bethesda, MD, USA.<br>Mice homozygous for the RhoP23H knock-in mutation [B6.129S6(Cg)-Rhotm1.1Kpal/J] were obtained from the Jackson Laboratories.<br>Nrl.GFP/+, RhoP23H/+ mice were bred by crossing RhoP23H/P23H knock-in with homozygous Nrl.GFP transgenic mice.<br>Both male and female mice were used throughout and ages of mice for specific experiments are stated throughout the manuscript.
Wild animals	No wild animals were used in this study.
Field-collected samples	No field collected samples were used in this study.
Ethics oversight	All animal procedures were conducted in accordance with the Animals (Scientific Procedures) Act 1986, UK and with the Association for Research in Vision & Ophthalmology (ARVO) statements on the care and use of animals in ophthalmic research under a UK Home Office Personal and Project License.

Note that full information on the approval of the study protocol must also be provided in the manuscript.
